# Scoring systems of metabolic syndrome and prediction of cardiovascular events: A population based cohort study

**DOI:** 10.1002/clc.23827

**Published:** 2022-04-14

**Authors:** Nima Motamed, Hossein Ajdarkosh, Mohammad Hadi Karbalaie Niya, Mahshid Panahi, Behzad Farahani, Nader Rezaie, Mehdi Nikkhah, Amir H. Faraji, Gholamreza Hemmasi, Dhayaneethie Perumal, G. Hossein Ashrafi, Fahimeh Safarnezhad Tameshkel, Esmaeel Gholizadeh, Mahmoodreza Khoonsari, Farhad Zamani

**Affiliations:** ^1^ Department of Social Medicine Zanjan University of Medical Sciences Zanjan Iran; ^2^ Gastrointestinal and Liver Diseases Research Center Iran University of Medical Sciences Tehran Iran; ^3^ Department of Cardiology Iran University of Medical Sciences Tehran Iran; ^4^ Department of Pulmonology, Firouzgar Hospital Iran University of Medical Sciences Tehran Iran; ^5^ Faculty of Science Engineering and Computing, Kingston University Kingston UK; ^6^ Cancer Theme SEC Faculty Kingston University London UK

**Keywords:** cardiovascular events, metabolic syndrome scoring system, structural equation modeling

## Abstract

**Background and Aims:**

Continuous scoring systems were developed versus traditional dichotomous approaches to define metabolic syndrome. The current study was carried out to evaluate the ability of scoring systems to predict fatal and nonfatal cardiovascular events.

**Materials and Methods:**

The data of 5147 individuals aged 18 years or more obtained from a population‐based cohort study were analyzed. The occurrence of atherosclerotic cardiovascular disease (ASCVD) in the period of 7 years follow‐up was considered as the associated outcome. Joint Interim Statement (JIS) definition, as a traditional definition of metabolic syndrome (MetS), and two versions of MetS scoring systems, based on standardized regression weights from structural equation modeling (SEM) and simple method for quantifying metabolic syndrome (siMS) were considered as potential predictors.

**Results:**

The scoring systems, particularly, based on SEM, were observed to have a significant association with composite cardiovascular events (HR = 1.388 [95% CI = 1.153–1.670], *p* = .001 in men and HR = 1.307 [0.95% CI = 1.120–1.526] in women) in multiple Cox proportional hazard regression analyses, whereas the traditional definition of MetS did not show any significant association. While both two scoring systems showed acceptable predictive abilities for cardiovascular events in women (MetS score based on SEM: area of under curve [AUC] = 0.7438 [95% CI = 0.6195–0.7903] and siMS: AUC = 0.7207 [95% CI = 0.6676–0.7738]), the two systems were not acceptable for identifying risk in men.

**Conclusion:**

Unlike the dichotomous definition of MetS, the scoring systems showed an independent association with cardiovascular events. Scoring systems, particularly those based on SEM, may be useful for the prediction of cardiovascular events in women.

## 1 INTRODUCTION

Cardiovascular diseases (CVD) are considered as the leading cause of death worldwide.^1^ Based on a recent data, CVD are the causes of one‐third of all deaths worldwide.^2^ The age standardized deaths related to CVD have reduced, particularly in high‐income countries by preventive measures and high‐quality interventions in spite of increase in absolute CVD deaths worldwide.^3^, ^4^ Despite a large decrease in the burden of CVD in the United States, the huge disparities in the total burden of CVD among different US states can be attributed to the differences in exposure to some modifiable risk factors.^5^ Some of these well‐known and modifiable cardiovascular risk factors including high blood pressure, abdominal obesity, high fasting blood sugar (FBS), low high‐density lipoprotein (HDL) levels, and obesity are used to define metabolic syndrome (MetS). A cluster of these multiple cardio‐metabolic abnormalities is defined as metabolic syndrome.^6^, ^7^, ^8^ Hence, a high association between the MetS and CVD is expected.^9^, ^10^, ^11^, ^12^, ^13^ As the prevalence of chronic diseases rises globally, it is important to identify individuals at greater risks of disease progression by evaluating their MetS statuses. However, this assessment of MetS severity and changes over time is challenging given the traditional dichotomous nature of the components that define MetS. Even a negligible change of one of the components can lead to classifying a person as having MetS or not. The limitations of the dichotomous MetS definition and its lack of universality have prompted the consequent development of continuous scoring systems with different approaches.^14^, ^15^, ^16^, ^17^ Even then, most available scoring equations have been formulated based on the western populations. On the other hand, cardiovascular risk assessment tools such as Systematic Coronary Risk Evaluation (SCORE) equations, Framingham general cardiovascular risk profile in primary care settings and American College of Cardiology/American Heart Association (ACC/AHA) risk prediction tool also have their own limitations.^18^, ^19^, ^20^ For instance, related models mostly are based on data that have already become out of date regarding the changes in preventive interventions and changes in the rate of endpoints.^21^, ^22^ Furthermore, these models mostly were developed in western countries and validated in small populations. As a result, the current study was conducted to evaluate the ability of the metabolic scoring systems, specific to attributes of the Iranian population, to predict the fatal and nonfatal CVD events.

## 2 METHODS

### 2.1 Study population

**Figure 1:**
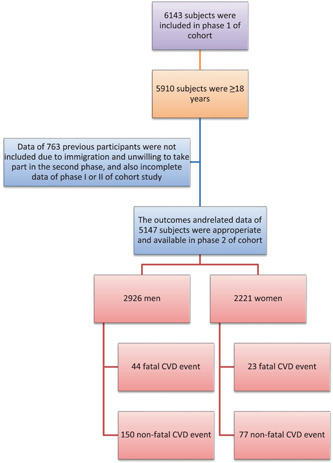
A schematic view of the study population in Phase I and Phase II of the cohort study. CVD, cardiovascular disease.

The present cohort study was carried out in two phases: Phase I in 2009–2010 and Phase II in 2016–2017. The study was performed on the population of Amol city, a relatively populated city in the central area of northern Iran. The sampling frame of our initial cohort study was based on the data from Health houses where an exact sampling frame is obtainable due to primary healthcare services delivered in these sittings. We divided the sampling frame into 16 strata based on gender and age groups, including 10–19, 20–29, 30–39, 40–49, 50–59, 60–69, 70–79, and 80–89 years. The size of each stratum in the sampling frame was calculated in proportion to the size of population in the same stratum using a stratified probability proportion sampling strategy. After 7 years, participants from the previous study in Phase I were invited again to participate in Phase II of the study. Since 2009–2010 up to the beginning of Phase II in 2016–2017, the study participants were annually contacted to collect related information about probable outcomes. A schematic view of the study population is shown in Figure 1.

The comprehensive evaluations of Phase II of our cohort started in 2016 and continued in 2017. This included a detailed evaluation of demographic, anthropometric, and laboratory data in addition to providing the related outcomes of people based on associated medical documents. It is worth noting that we confirmed and modified, where necessary, the related outcomes based on the data from various authentic sources, such as valid documented data from hospitals, clinics, physician offices, and also medical records, particularly if there was any inconsistency in the findings.

### 2.2 Anthropometric parameters and blood pressure measurements

Participants’ heights were measured via a nonstretchable meter. This was performed when they were in the upright position with a small gap between the legs (usually 10 cm) and their back of heads, shoulder blades, buttocks, and heels in contact with the wall. Also, participants’ weights were measured using a calibrated scale with a precision of 100 g. Waist circumference (WC) was measured by trained staff from the narrowest point between the lower borders of the rib cage and the iliac crest using a nonstretchable tape measure. Participants’ blood pressure was measured by trained staff using a mercury sphygmomanometer after the participant sat on a chair for a minimum of 5 min of physical inactivity in a quiet room. After inflation of the cuffs 20–30 mmHg above the point of disappearance of the radial pulse, the cuffs were deflated at a rate of approximately 2–3 mmHg. Thus, the appearance and disappearance of Korotkoff sounds were considered as the systolic (SBP) and diastolic (DBP) blood pressures, respectively. The average of two measurements of blood pressures for each participant was considered as the associated blood pressure of the participant.

### 2.3 Biochemical measurements

Totally, 10 ml of whole blood was obtained from each participant using a serum separator tube (SST; tiger top tube). Following 12‐h fasting, FBS, and lipid profiles were evaluated. All the tests, including FBS, triglycerides (TG), HDL, LDL, and cholesterol were enzymatically assessed using the BS200 Auto analyzer (Mindray).

### 2.4 Metabolic syndrome

Dichotomous version of metabolic syndrome definition was defined based on Joint Interim Statement (JIS).^23^

### 2.5 Outcomes

The occurrence of atherosclerotic cardiovascular disease (ASCVD) was considered the associated outcome of the present study. The definition of ASCVD was considered as a history of nonfatal acute myocardial infarction and ischemic heart disease death and fatal and nonfatal cerebrovascular accident.^24^

The outcome data were annually collected from the participants, although a comprehensive assessment in Phase II of the cohort was performed, too. Outcomes were obtained from the records of hospital admissions, including myocardial infarction or other CVD events, angiographically proven coronary heart diseases, history of percutaneous coronary interventions, and cerebrovascular diseases (from 2009–2010 to 2016–2017). All self‐reported data were confirmed or adjusted based on direct observation of valid documentations of medical records. The death certificates for fatal CVD events and hospital discharge records were also evaluated and verified. We actively contacted the medical centers where the patients were admitted, if a medical record did not seem to be correct. Each inconsistent finding between the outcomes of the comprehensive assessment and the annually obtained outcomes data were modified based on valid documented data.

A 12‐lead electrocardiogram (ECG) was performed by trained nurses for all participants who participated in Phase II of the cohort study. Consequently, if any ECG abnormality was seen in the participants from the Phase II cohort, their abnormality was not included as the outcome, the participant was comprehensively examined by the internist of our team, and was also referred to an expert cardiologist to rule out any silent CVD events in the follow‐up periods. Finally, all associated outcomes were confirmed by the internist of our cohort study team.

### 2.6 Statistical analysis

We conducted a confirmatory factor analysis (CFA) using maximum likelihood estimation to evaluate factor validity of MetS in addition to computing the standardized regression weights of related observed variables. Thus, a single‐factor model was conducted in which MetS was considered as the latent variable and the components of MetS were considered as the observed variables. To enable a comparison of the MetS single‐factor model and the simple method for quantifying metabolic syndrome (siMS) score, we considered the same observed variables included in siMS score in our CFA models. These variables included the SBP, FBS, TG, HDL, and WC as observed variables. However, a model based on SBP, FBS, WC, and the natural logarithm of (TG/HDL) obtained the best‐fit indices in structural equation modeling (SEM) models. Figure S1 shows a scheme of the indices of the SEM model used in our study.

The related fit indices were standardized root mean square residual (SRMR), Comparative Fit Index, Goodness of Fit Index (GFI), and root mean square error of approximation (RMSEA). Despite lack of a general agreement about the appropriate values of these fit indices, some thresholds for a good model fit were suggested for these indices, as follows: GFI ≥ 0.95, RSMEA < 0.08, SRMR < 0.08 (the values greater than 0.1 denote a poor fit), AGFI = 0.95, and CFI ≥ 0.95. However, according to Hu and Bentler, an RSMEA = 0.06 can be considered as an appropriate value of model fit.^25^, ^26^, ^27^, ^28^ The regression weights of the observed variables were utilized to build the MetS score models. A related equation is shown below:

$$\begin{array}{l} \mathrm{MetS}\;\mathrm{Score}\;(\mathrm{based}\;\mathrm{on}\;\mathrm{SEM})\\ \;=\beta_{\mathrm{WC}}\times \mathrm{WC}\;(\mathrm{cm})+\beta_{\mathrm{SBP}}\times \mathrm{SBP}(\mathrm{mmHg})+\beta_{\ln\left(\frac{\mathrm{TG}}{\mathrm{HDL}}\right)}\times \;\ln\left(\frac{\mathrm{TG}\;(\mathrm{mg}/\mathrm{dl})}{\mathrm{HDL}\;(\mathrm{mg}/\mathrm{dl})}\right)\\ \;+\;\beta_{\mathrm{FBS}}\times \mathrm{FBS}\;(\mathrm{mg}/\mathrm{dl}), \end{array}$$

where the *β*s are considered the related standardized regression weights. To obtain the MetS score (based on SEM), the related standardized regression weights of SEM were inserted instead of associated coefficients (*β*s) in the above formula. The MetS scores were calculated based on the above formula for all participants. CFA was conducted in men and women separately, and different standardized regression weights were obtained in men and women based on the CFA, separately. The SEM was conducted in AMOS IBM SPSS 21.

siMS was calculated based on the following formula^15^:

$$\begin{array}{l} \mathrm{siMS}\;\mathrm{score}=2\times \frac{\mathrm{WC}\;(\mathrm{cm})}{\mathrm{Height}\;(\mathrm{cm})}+\frac{\mathrm{FBS}\;(\mathrm{mg}/\mathrm{dl})}{100}+\frac{\mathrm{TG}\;\left(\mathrm{mg}/\mathrm{dl}\right)}{150}+\frac{\mathrm{SBP}\;(\mathrm{mmHg})}{130}+\frac{\mathrm{HDL}\;(\mathrm{mg}/\mathrm{dl})}{40\;\mathrm{in}\;\mathrm{men}\;\mathrm{and}\;50\;\mathrm{in}\;\mathrm{women}}.\\ \; \end{array}$$

To determine the predictive ability of two versions of MetS scores, receiver operating characteristic (ROC) analyses were conducted. Thus, the related MetS scores were considered as the classification variables and CVD events were considered as the reference variable (*Y *= 1 for the occurrence of outcomes in the participants during the 7 years of follow‐up and *Y *= 0 for the nonoccurrence of outcomes in this period). The related outcomes were fatal CVD events, nonfatal CVD events, and composite CVD events (summation of fatal and nonfatal CVD events). The ROC analyses were separately conducted on these three types of CVD events in men and women. The predictive abilities were reported based on the results of the areas under curves (AUCs). Thus, AUCs were calculated based on plotting the sensitivities of infinite decision thresholds of the classification variables versus their false‐positive rates in the prediction of related CVD outcomes. An AUC of >0.5 to <0.7 was considered as poor ability, AUC >0.7 to <0.8 as acceptable ability, AUC >0.8 to <0.9 as an excellent ability, and AU >0.9 to <1.0 was considered as outstanding ability. Also, an AUC = 0.5 indicated “no” ability while an AUC = 1 indicated a “perfect” ability. These considerations were based on Hosmer and Lemeshow's guidelines.^29^

In ROC analyses, all statistical analyses were conducted using Stata software, version 12 (STATA Corp). The rocreg (ROC regression) package of Stata software was used to obtain the AUCs and the plots of ROC curves.

The simple and multiple Cox hazard regression proportion models were performed where the time of occurrence of fatal and nonfatal CVD events was considered as related outcomes. In multiple hazard regression proportion models, the age, LDL‐C level, smoking status, and DBP were entered in addition to the MetS scores. We conducted the Cox regression models for the two versions of MetS scores (their *Z*‐scores) separately for both men and women. The Cox models were also performed on CVD events for dichotomous version of MetS definition (based on JIS definition). The related hazard ratios (and their 95% confidence intervals [CI]) were reported. The multiple hazard regression proportion models were conducted using SPSS, version 21 (Chicago Statistical Software, Inc.).

## 3 RESULTS

Table 1 shows basic characteristics of the study population based on sex. While the mean age (*p *= .003), weight (*p* < .001), DBP (*p *= .022), and SBP (*p* < .001) were significantly higher in men, BMI (*p* < .001), FBS (*p *< .000), cholesterol (*p* < .001), HDL‐C (*p* < .001), and LDL‐C (*p* < .001) were significantly lower in men as compared with those in women. No significant difference in terms of mean WC and TG was observed between men and women; however, both the MetS and siMS scores were significantly higher in women (*p*'s <.001).

**Table 1:** Basic characteristics of the study population of primary phase of cohort

Variables	Mean ± SD		*p *value
	Men (*n* = 2926)	Women (*n* = 2221)	
Age (year)	44.46 ± 16.73	43.25 ± 15.25	0.003
Weight (kg)	76.86 ± 15.08	72.71 ± 14.36	<0.001
Hip circumference (cm)	100.88 ± 8.36	106.69 ± 10.92	<0.001
WC (cm)	90.82 ± 12.43	91.49 ± 13.40	0.068
BMI (kg/m^2^)	26.53 ± 4.61	29.66 ± 5.67	<0.001
DBP (mmHg)	76.59 ± 12.61	75.76 ± 13.20	0.022
SBP (mmHg)	117.25 ± 15.55	115.27 ± 17.70	<0.001
FBS (mg/dl)	98.53 ± 29.86	103.80 ± 41.33	<0.001
TG (mg/dl)	144.80 ± 91.86	141.75 ± 98.44	0.065
Cholesterol (mg/dl)	178.64 ± 41.85	188.69 ± 43.04	<0.001
HDL‐C (mg/dl)	43.49 ± 11.58	46.37 ± 12.11	<0.001
LDL‐C (mg/dl)	104.95 ± 30.94	109.49 ± 31.41	<0.001
MetS score (based on SEM)	153.55 ± 17.64	163.77 ± 24.92	<0.001
siMS score	2.83 ± 0.918	3.12 ± 1.07	<0.001

Abbreviations: BMI, body mass index; DBP, diastolic blood pressure; FBS, fasting blood sugar; HC, hip circumference; HDL‐C, high‐density lipoprotein cholesterol; LDL‐C, low‐density lipoprotein cholesterol; SBP, systolic blood pressure; TG, triglyceride; WC, waist circumference.

Significance level for the difference between men and women was considered *p* < .05.

**Figure 2:**
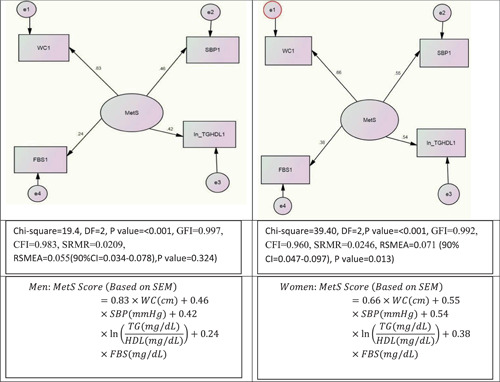
The results of SEM models and related MetS scoring systems. CFI, Comparative Fit Index; CI, confidence interval; FBS, fasting blood sugar; GFI, Goodness of Fit Index; ln, natural logarithm; MetS, metabolic syndrome; RMSEA, root mean square error of approximation; SBP, systolic blood pressure; SEM, structural equation modeling; SRMR, standardized root mean square residual; TGHDL, TG (triglyceride) to HDLc (high‐density lipoprotein cholesterol) ratio; WC, waist circumference.

Figure 2 shows the results of fit indices and the standardized regression weights of the SEM models in men and women, separately. The related formulas of scoring models were built based on the standardized regression weights of these SEM models.

The predictive ability and CIs of the two versions of MetS scores for CVD events are shown in Table 2. In men, the predictive ability of MetS score based on SEM was acceptable for fatal CVD events (AUC = 0.7049 [95% CI = 0.6195–0.7903]), while in women, excellent predictive abilities were obtained by both scoring systems for this type of events (MetS scores based on SEM: AUC = 0.8277 [95% CI = 0.7614–0.8940] and siMS systems: AUC = 0.8447 [95% CI = 0.7986–0.9808]). For nonfatal CVD events in women, only the MetS score based on SEM showed an acceptable ability (AUC = 0.7129 (95% CI = 0.6504–0.7754]). When considering composite CVD events, both two scoring systems showed acceptable predictive abilities in women (MetS score based on SEM: AUC = 0.7438 [95% CI = 0.6195–0.7903] and siMS: AUC = 0.7207 [95% CI = 0.6676–0.7738]), but poor abilities were found in men (AUC > 0.5–<0.7).

**Table 2:** The predictive ability of two versions of MetS scores for CVD events

Outcomes	Sex	AUC (95% CI)		*p *value
		MetS score (based on SEM)	siMS score	
Fatal CVD events	Men	0.7049 (0.6195–0.7903)	0.6121 (0.5246–0.6995)	<0.001
	Women	0.8277 (0.7614–0.8940)	0.8447 (0.7986–0.9808)	0.581
Nonfatal CVD events	Men	0.6232 (0.5743–0.6722)	0.5720 (0.5243–0.6197)	0.005
	Women	0.7129 (0.6504–0.7754)	0.6779 (0.6151–0.7408)	0.117
Composite CVD events	Men	0.6458 (0.6041–0.6876)	0.5834 (0.5416–0.6252)	0.001
	Women	0.7438 (0.6906–0.7971)	0.7207 (0.6676–0.7738)	0.216

Abbreviations: AUC, area under receiver operating characteristic (ROC) curve; CI, confidence interval; CVD, cardiovascular disease; MetS, metabolic syndrome; SEM, structural equation modeling.

Significance level for the difference between scoring systems was considered *p* < .05.

Additionally, in women, there was no statistical difference between the two scoring systems for fatal, nonfatal, and composite CVD events with both scores being excellent and acceptable predictors for fatal and composite events, respectively (Table 2 and Supplementary Figure 2). However, the degree of predictive ability for the MetS Score (based on SEM) was superior for nonfatal CVD events as compared with the siMS score (poor ability). On the other hand, in men, MetS score (based on SEM) was statistically superior to siMS for all events (*p*<.05 for fatal, nonfatal, and composite). Yet, all of the MetS scores (based on SEM and siMS) in men, aside from the MetS score based on SEM for fatal CVD events which showed marginal acceptable prediction, were classified as poor according to the AUC considerations based on Hosmer and Lemeshow's guidelines^29^ (Table 2 and Figure S2).

Table 3 shows the results of simple and multiple Cox proportional hazards regression analyses where the time of the occurrence of CVD events (fatal, nonfatal, and composite) was considered as the associated outcome. In simple Cox analysis, the MetS score (based on SEM) and siMS showed a significant association with fatal, nonfatal, and composite CVD events in men and women. While the traditional definition of MetS did not show any association with CVD events in men, a significant association was detected in women for all types of the events. In multiple Cox proportional hazards regression analysis, no independent association was detected between CVD events and the traditional dichotomous definition of MetS. However, a significant independent association was detected between the two versions of scoring systems and all types of CVD events except for nonfatal CVD events and siMS in men.

**Table 3:** Simple and multiple Cox regression proportional analyses on the time of the occurrence of CVD events in which the MetS scores were the predictor

Gender	Scoring system	CVD outcomes	Simple		Multiple	
			HR (95% CI)	*p *value	HR (95% CI)	*p *value
Men	MetS score (based on standardized regression weights of SEM)	Fatal	2.286 (1.765–2.961)	<.001	1.778 (1.268–2.493)	<.001
		Nonfatal	1.586 (1.337–1.882)	<.001	1.293 (1.037–1.612)	.022
		Composite	1.749 (1.516–2.018)	<.001	1.388 (1.153–1.670)	.001
	siMS	Fatal	1.505 (1.179–1.920)	.001	1.503 (1.113–2.028)	.008
		Nonfatal	1.243 (1.065–1.450)	.006	1.108 (0.929–1.321)	.256
		Composite	1.306 (1.145–1.488)	<.001	1.171 (1.006–1.363)	.042
	MetS definition based on JIS	Fatal	1.405 (0.733–2.692)	.306	1.111 (0.555–2.224)	.767
		Nonfatal	1.380 (0.978–1.948)	.067	1.096 (0.752–1.596)	.633
		Composite	1.386 (1.022–1.879)	.036	1.080 (0.776–1.504)	.647
Women	MetS score (based on standardized regression weights of SEM)	Fatal	1.830 (1.484–2.257)	<.001	1.472 (1.092–1.983)	.011
		Nonfatal	1.569 (1.378–1.786)	<.001	1.276 (1.062–1.533)	.009
		Composite	1.632 (1.462–1.821)	<.001	1.307 (1.120–1.526)	.001
	siMS	Fatal	1.635 (1.375–1.943)	<.001	1.656 (1.208–2.270)	.002
		Nonfatal	1.429 (1.261–1.620)	<.001	1.247 (1.019–1.526)	.032
		Composite	1.485 (1.342–1.644)	<.001	1.325 (1.120–1.568)	.001
	MetS definition based on JIS	Fatal	25.40 (3.423–188.402)	.002	7.700 (1.002–59.145)	.050
		Nonfatal	2.561 (1.528–4.291)	<.001	1.095 (0.616–1.948)	.758
		Composite	3.602 (2.227–5.825)	<.001	1.416 (0.837–2.397)	.195

Abbreviations: CVD, cardiovascular disease; CI, confidence interval; HR, hazard ratio; JIS, Joint Interim Statement MetS, metabolic syndrome; SEM, structural equation modeling.

Significance level was considered *p* < .05.

## 4 DISCUSSION

We evaluated the association between CVD events and MetS by applying simple and multiple Cox proportional hazards models. Thus, two versions of MetS scores and MetS definition, based on JIS,^23^ were considered as potential predictors. According to our results, while the scoring systems showed a significant association with CVD events in multiple Cox models removing potential mediators, including age, DBP, LDLc, and smoking status, we found that the MetS, according to the definition of JIS, did not have any association in this aspect. In this regard, consideration of the continuous values of risk components and the weightings thereof in the scoring system showed an improvement in its ability to predict CVD relative to the use of the MetS dichotomous classification. The dichotomous approach in the definition of MetS ignores the severity of individual components of MetS and thus a large part of important information in this syndrome will be missed. Consequently, it is likely that the association between this syndrome and CVD events is underestimated. On the other hand, in the dichotomous approach, even a negligible change of one of the components can lead to classifying a person as having MetS or not. Thus, two patients with an almost similar nature of the related CVD risk factor may be classified into different statuses of MetS (has/has not). As a result, a potential association may not be identified. The limitations of the dichotomous MetS definition and the lack of universal agreement in this type of definition make it necessary to develop other types of definitions, particularly continuous scoring systems.^14^, ^15^, ^16^, ^17^

The present study also compared the predictive ability of two versions of the scoring systems of the MetS in the prediction of fatal and nonfatal CVD events in a cohort study with the follow‐up period of 7 years. Overall, the results showed that the scoring system based on SEM had a better predictive ability compared with the siMS scoring system. The ability of Mets scoring systems was excellent for the prediction of fatal CVD events in women. In men, while scoring system based on SEM showed an acceptable ability for the prediction of fatal CVD events, the ability of the siMS to predict the same events was considered poor. As for nonfatal events, both scoring systems showed poor predictive abilities in men. However, for women, the scoring system based on SEM showed an acceptable ability for the prediction of nonfatal CVD events relative to the siMS scoring of poor predictive ability. Our results in women revealed that Mets score based on SEM had an ability approximately similar to that of MetS score, as evaluated by Yang et al.^16^ in the Kazakhs population in the far west of China for composite CVD events applying age in addition to MetS components. Yang et al.^16^ showed that the ability of MetS risk score in the prediction of CVD events was not acceptable (AUC = 0.647). However a different approach was applied to develop the scoring system by Yang et al.^16^ Moreover, when the authors incorporated age into the model, an acceptable ability for the prediction of CVD events was obtained. Age may therefore play a role in predicting the development of CVD using MetS risk scores. Our study did not apply age in the scoring system based on SEM as applying age did not fit appropriately with our data in the single‐factor model. Age is one of the most important prognostic variables to develop the CVD events.^24^

On the other hand, several cardiovascular risk assessment tools have been developed to predict the CVD events. In this regard, Kavousi et al.^30^ showed that SCORE equations (for the prediction of fatal CVD events) had an acceptable ability for fatal CVD events both in men and women. In the male population of Kavousi et al. study, the abilities of the American College of Cardiology/American Heart Association (ACC/AHA) and Adult Treatment Panel III (ATPIII) models were found to be higher than that of MetS scores based on SEM in the male population of our study for the prediction of composite CVD events. However, the MetS scores based on SEM showed a higher prediction ability in our women compared with the cardiovascular risk assessment tools used in the women population of Kavousi et al. study. This may emphasize gender as an important variable in CVD prediction with the MetS scoring systems. However, the difference in the mean age of the population included in the two studies should cause the comparison of the results of the two studies to be interpreted with more caution. Although the MetS scoring system based on SEM showed a comparable result with those of risk assessment tools, in which age was utilized to estimate the 10‐year CVD risks, the development of risk MetS scores applying this approach and considering age as a prognostic factor may improve the ability of these MetS scores.

Our study had certain limitations, which should be taken into consideration before any generalization. Although a comprehensive evaluation was performed to obtain the related outcomes, the lack of ECG data in the Phase I of the cohort may have resulted in the possibility that some participants may have developed silent CVD events during follow‐up and that these outcomes would have been excluded from our data. However, our evaluation did detect 21 cases of ECG changes that were not included as outcomes in our study. These participants were evaluated by the team internist and also referred to an expert cardiologist to determine their possible outcomes. Furthermore, a scenario analysis including the outcomes of these participants did not show any significant and reportable changes. Also, in the present study, we did not consider the MetS scores based on age due to nonfitting of the single factor of the model wherein age was also applied. As mentioned previously, age is a strong prognostic factor for the development of cardiovascular events and thus the predictive ability of models based on age can be improved. Nevertheless, our study provided insight on parameters related to the prediction of CVD, particularly the ability of the scoring systems in women and its implications for public health initiatives in northern Iran populations.

## 5 CONCLUSION

It was found that the scoring systems showed a stronger association with CVD events compared with the definition of the Mets. Both scoring systems showed an excellent ability to predict fatal CVD in women and acceptable ability in composite CVD events irrespective of gender. Overall, the ability of the scoring system based on SEM was superior to siMS. Since the early prediction of CVD plays an important role in decreasing CVD incidence and developing effective target population‐based strategies, the scoring systems of MetS can be valuable in this instance.

## CONFLICTS OF INTEREST

The authors declare no conflicts of interest.

## Supporting information

Supplementary information.

## Data Availability

The data supporting this study's findings are available from the corresponding author upon reasonable request.
