# Supplementary material for: Scoring systems of metabolic syndrome and prediction of cardiovascular events: A population based cohort study
Source: Clin Cardiol. 2022 Apr 14;45(6):641–9. doi: 10.1002/clc.23827 (PMC9175260; doi:10.1002/clc.23827)
Supplement: Supplementary file 1 — Supplementary information. [file CLC-45-641-s001.docx]

**supplementary Figure 1: A scheme of the indices of the SEM model applied in this study.** FBS: Fasting blood sugar, ln: Natural logarithm, MetS: Metabolic syndrome, TGHDL: TG (triglyceride) to HDLc (High density lipoprotein cholesterol) ratio, SBP: Systolic blood pressure, and WC: waist circumference.

**supplementary Figure 2: Comparison of ROC curves to determine the predictive ability of the MetS (blue curve) and siMS (red curve) scores for fatal, Non-fatal and composite CVD events for men and women in phase II of the cohort study.** AUC: Area under receiver operating characteristic (ROC) curve, CVD: Cardiovascular disease, MetS: Metabolic syndrome, ROC: receiver operating characteristic, and SEM: Structural equation modeling.
